# Nephrotoxicity of Herbal Medicine and Its Prevention

**DOI:** 10.3389/fphar.2020.569551

**Published:** 2020-10-15

**Authors:** Xiaofen Xu, Ruyi Zhu, Jialiang Ying, Mengting Zhao, Xin Wu, Gang Cao, Kuilong Wang

**Affiliations:** ^1^School of Pharmacy, Zhejiang Chinese Medical University, Hangzhou, China; ^2^School of Pharmacy, Guizhou University of Traditional Chinese Medicine, Guiyang, China

**Keywords:** herbal medicine, nephrotoxicity, toxic components, toxicity mechanism, prevention strategies

## Abstract

Herbal medicine (HM) has been widely used to treat diseases for thousands of years and has greatly contributed to the health of human beings. Many new drugs have been developed from HM, such as artemisinin. However, artemisinin has adverse effects, such as renal toxicity. In 1993, a study conducted in Belgium reported for the first time that the root extracts of Aristolochia obliqua S. M. Hwang led to progressive interstitial renal fibrosis. The nephrotoxicity of HM has attracted worldwide attention. More than 100 kinds of HM induce renal toxicity, including some herbs, animal HMs, and minerals. This paper aimed to summarize the HM compounds that cause nephrotoxicity, the mechanisms underlying the toxicity of these compounds, biomarkers of renal injury, and prevention strategies. These findings provide a basis for follow-up studies on the prevention and treatment of HM nephrotoxicity.

## Introduction

With the extensive use and deepening pharmacological research on HM, the adverse effects of HMs have also been determined. HM differs from western medicine, its side effects are considered slight, and it can be taken for a long time or at a large dose. However, in recent years, reports of adverse reactions caused by HM and its preparations have increased every year. Nephrotoxicity is one of the main toxicities of HMs. In the 1960s, some scholars first reported the cases of acute renal failure caused by the administration of *Aristolochia manshuriensis* Kom in China, after which relevant reports also appeared abroad (Wu, 1964). In 1993, Belgian scholars reported that nine European women suffered from renal failure after taking weight-loss capsules containing Chinese herbal medicine *A. obliqua* S. M. Hwang; this condition is called “Chinese herb nephropathy (CHN)” (Vanherweghem et al., 1993).

Besides aristolochic acid, many other HM components cause renal toxicity. Severe renal tubular lesions have been found in renal puncture examinations of patients with kidney disease caused by *Tripterygium regelii* Sprague. et. Takeda. The lesions are accompanied by obvious inflammatory cell infiltration, degeneration, and necrosis of renal tubular epithelial cells (Luo et al., 2018b). The administration of a large amount of *Glycyrrhiza uralensis* Fisch. caused rhabdomyolysis and acute renal injury. Many kinds of animal HMs can cause renal toxicity, such as *Scolopendra subspinipes mutilans* L. Koch, fish gall, and *Mylabris*. Fish gall can cause the swelling and necrosis of renal tubular epithelial cells and medullary edema. *Hirudo nipponica* Whitman and *S. subspinipes mutilans* L. Koch may lead to hemolytic reaction, aggravating the damage to renal function and eventually leading to massive hematuria. Realgar, a mineral Chinese medicine, directly injures the glomerulus. In summary, according to research statistics, more than 100 kinds of HM have toxic effects on the kidney. This article focuses on the kidney toxicity of HMs and their possible mechanisms and summarizes the strategies for preventing HM nephrotoxicity.

Statistics state that more than 100 kinds of HM preparations can cause kidney damage. With the popularization of Chinese culture, HM has also become gradually known and exported to South Korea, Japan, India, Germany, and other European countries, thus playing a huge role in the world medical system (Wang, 2013). Therefore, improving the understanding of HM drugs with renal toxicity effects has become an urgent problem. In this paper, HM drugs with renal toxicity were discussed in accordance with their types of toxic components. Moreover, the causes of kidney injury and the toxicity mechanisms of components were introduced in detail. The measures to reduce renal toxicity were elaborated upon at length on the basis of modern science and technology. This elaboration is conducive to follow-up studies on the prevention and treatment measures of HM and the safety of clinical medication.

## Compounds Liable for Nephrotoxicity and Representative HM

### Organic Acids

Organic acid is an organic compound (excluding amino acid) that contains a carboxyl group. This compound widely exists in plants. Aristolochic acid (1) is the most representative nephrotoxic component among the HM organic acids, and its representative HMs are *A. manshuriensis* Kom, *A. obliqua* S. M. Hwang, and *A, debilis* Sieb. Et Zucc. Aristolochic acid can cause necrosis and apoptosis of renal tubular epithelial cells.

### Alkaloids

Alkaloid is a nitrogen-containing organic compound that exists in nature and is obtained from plants. It is a toxic component of many HMs, such as matrine (2) and genistein in *Sophora flavescens* Ait., glycoside alkaloid in *Alisma orientale* (Sam.) Juzep., arecoline (3) in *Areca catechu L*., and colchicine (4) detected in *Gremastra appendiculata* (D. Don) Makino. Other examples are Tetrandrine (5), Ephedrine (6), Leonurine (7). Most types of nephrotoxic alkaloid are organic amines, pyrrolidines, pyridines, and isoquinolines.

### Terpenes and Lactones

Terpenoids and lactones are ubiquitous compounds in the plant kingdom. Terpenoids are derivatives with a general formula of (C_5_H_8_) _n_, oxygen content, and different saturation degrees. Lactone is an organic compound with one ester group formed by the esterification of one molecule. They have important physiological activities and are important resources for the research of natural products and the development of new drugs. This kind of nephrotoxic component includes cinnamaldehyde (8), thujone (9) and triptolide (10). Representative HMs include *Cinnamomum cassia* Presl*, Magnolia officinalis* Rehd. et Wils.*, Melia toosendan* Sieb.et Zucc., and *Leonurus japonicus* Houtt., and the nephrotoxicity of these substances is weaker than that of aristolochic acid. In clinical use, doctors often focus on its efficacy and ignore its toxicity. This substance can cause renal tubulointerstitial damage, the sufferers of which may show symptoms of uremia and proteinuria.

### Toxic Proteins

A virulent protein is obtained from a normal cellular protein, PrPc, which has been changed but does not have the function of a normal protein. It is the smallest known infectious medium with a single component. It is nearly only composed of protein, but without nucleic acid. It can strongly resist proteolytic enzymes. These toxic substances are obtained from protein components in animal and plant medicinal materials, such as trichosanthin in *Trichosanthes kirilowii* Maxim, histamine-like substance hemolytic protein in *S. subspinipes mutilans* L. Koch, and leech protein in *Hirudo nipponica* Whitman.

### Minerals

Most of the toxic components of minerals contain heavy metals, which include arsenic, mercury, and lead. The representative drugs are Ae_2_S_2_ in Realgar, HgS in cinnabaris, and Pb in Red lead. After taking these drugs in large quantities, heavy metals accumulate in the kidneys because of metabolic difficulties, resulting in kidney damage. For example, cinnabars accumulate in the kidney and lead to the apoptosis of renal tubular epithelial cells in the renal cortex.

### Flavonoid Glycosides

Flavonoid glycosides are widely found in plants and berries with strong antioxidant properties. Even low amounts of flavonoid glycosides cause kidney damage in HM. Some of these flavonoid glycosides come from dry mature seeds of *Ginkgo biloba*, the toxic component of which is ginkgo neuter. Flavonoid glycosides are a series of compounds formed by the interconnection of two benzene rings (A- and B-rings) with phenolic hydroxyl groups through the central three carbon atom. Their basic parent nucleus is 2-phenylchromogenic ketone.

### Saponins

Saponin is a plant glycoside that can form colloid solution and soap-like foam. The representative HMs containing saponins are *Xanthium sibiricum* Patr., *Bupleurum chinense* DC., *Clematis chinensis* Osbeck, and *Pulsatilla chinensis* (Bge.) Regel. In addition to *Pulsatilla chinensis* (Bge.) Regel, the toxic component of *C. chinensis* Osbeck is also anemonin (11). Long-term administration of *C. chinensis* Osbeck lead to acute renal tubular injury, necrosis, or chronic interstitial nephritis. Other components with nephrotoxicity are saikosaponin A (12), geniposide (13), esculentoside A (14). Saponins are glycosides composed of aglycones and sugars, glucuronic acids, or other organic acids. According to the known molecular structure of aglycones, the saponins of c-27 steroids are called steroids and those of triterpenoids are called triterpenoids.

**Figure 1:**
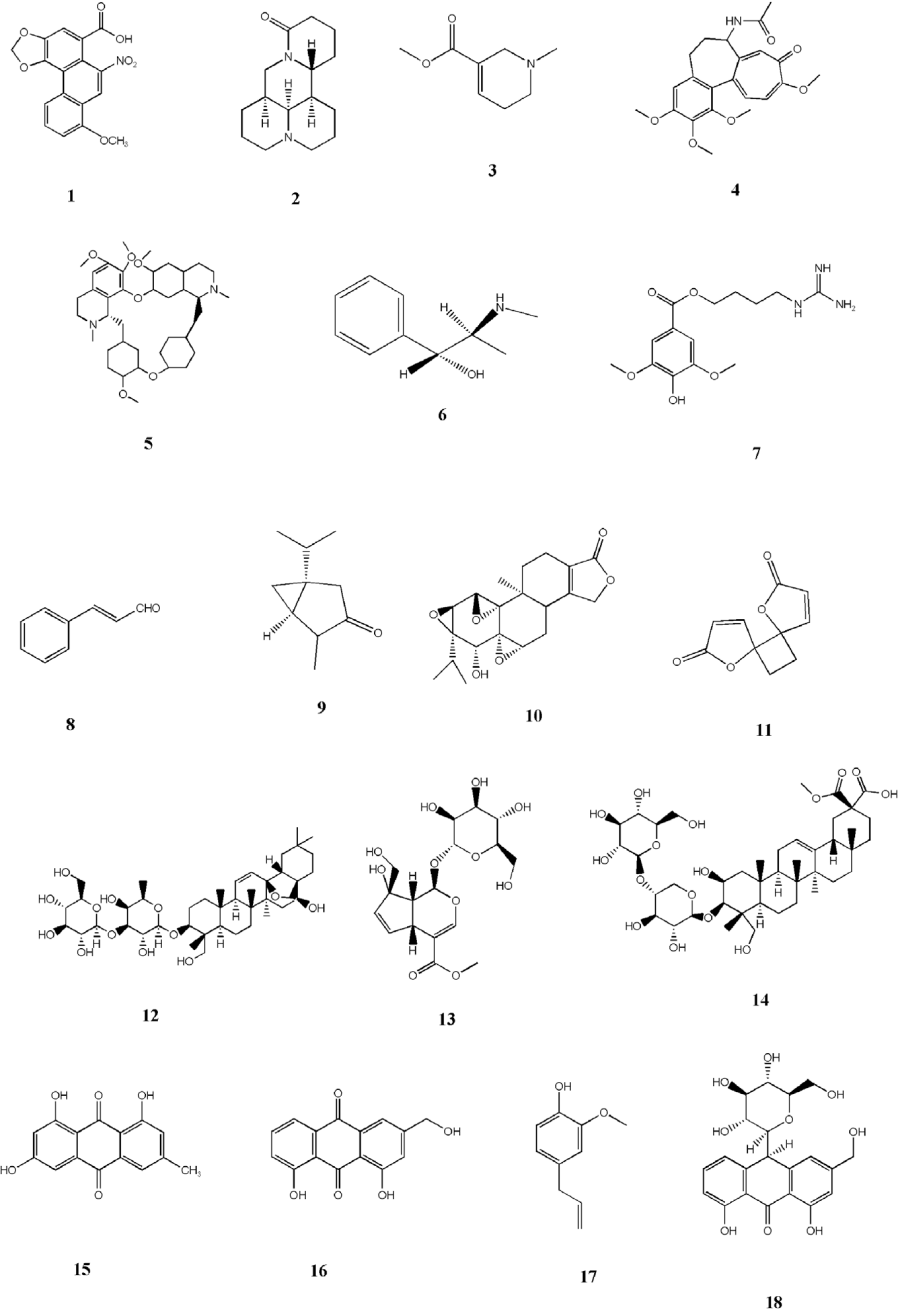
Representative nephrotoxic compounds structures.

According to statistics, more than 100 kinds herbs may cause kidney toxicity in HM, and their toxic components are not fully understood. In addition to the representative toxic compounds listed above, anthraquinones in *Polygonum cuspidatum* Sieb.et Zucc. are toxic components, and the main toxic components in rhubarb are emodin (15), aloe emodin (16) and other anthraquinones. The toxic component of *Eugenia caryophyllata* Thunb. is eugenol (17), which is an aromatic compound. Aloin (18) is a nephrotoxic component in *Aloe barbadmsis* Miller. The toxic components of fish gall, such as histamine or oxide, are complex. Other known nephrotoxic HMs such as *Schizonepeta tenuifolia* Briq*., Bolbostemma paniculatum* (Maxim.) Franquet, *Euphorbia pekinensis* Rupr., and *Eugenia caryophyllata* Thunb. These need further experimental analysis to determine their toxic components. **Table 1** shows the HM nephrotoxicity and their toxic ingredients. **Figure 1** shows the representative structure of nephrotoxic components.

**Table 1:** Chinese herbal medicines known to contain kidney toxicity components.

Latin name	English name	Nephrotoxiccompounds	References
*Aristolochiaceae*	Aristolochic	Aristolochic acid	(Lv et al., 2012)
*Aristolochia manshuriensis* Kom	Manshuriensis	Aristolochic acid	(Lv et al., 2012)
*Aristolochia obliqua* S. M. Hwang	Fangchi	Aristolochic acid	(Lv et al., 2012)
*Aristolochia debilis* Sieb. et Zucc.	Radix aristolochiae	Aristolochic acid	(Lv et al., 2012)
*Asarum heterotropoides* Fr. Schmidt var. Mandshuricum (Maxin.) Kitag.	Asarum sieboldii	Aristolochic acid	(Lv et al., 2012)
*Aristolochia cinnabarina* C. Y. Cheng et J. L. Wu	Root of Kaempfer Dutchmans pipe	Aristolochic acid	(Lv et al., 2012)
*Aristolochia mollissima* Hance.	Aristolochia	Aristolochic acid	(Lv et al., 2012)
*Sophora flavescens* Ait.	Sophora flavescens	Matrine	(Weng et al., 2018)
*Leonurus ja ponicus* Houtt.	Motherwort	Leonurine	(Huang and Sun, 2010)
*Arecacatechu* L.	Betelnutpalm Seed	Arecoline	(Zeng et al., 2015)
*Cinnamomum cassia* Presl	Cinnamon Bark	Cinnamaldehyde	(Zhang et al., 2019)
*Melia toosendan* Sieb.et Zucc.	Toosendan Fruit	Toosendanin	(Li et al., 2017)
*Tripterygium regelii* Sprague. et Takeda.	Tripterygium Root	Triptolide	(Xi et al., 2017)
*Trichosanthes kirilowii* Maxim.	Mongolian Snakegourd Root	Trichosanthin	(Jiao et al., 2014)
*Croton tiglium* L.	Croton	Crotin	(Jiao et al., 2014)
*Xanthium sibiricum* Patr.	Xanthium Fruit	Xanthotoxin	(Jiao et al., 2014)
*Ricinuscommunis*L.	Castor Seed	Ricin	(Jiao et al., 2014)
*/*	Realgar	Ae_2_S_2_	(Jiao et al., 2014)
*/*	Cinnabaris	HgS	(Jiao et al., 2014)
*Phytolacca acinosa* Roxb.	Pokeberry Root	Esculentoside A	(Yang X. et al., 2019)
*Gardenia jasminoides* Ellis	Gardenia	Geniposide	(Feng et al., 2016)
*Rheum palmatum* L.	Rhubarb	Emodin	(Hu et al., 2019)
*Pulsatilla chinensi*(Bge.)Regel	Anemone	Anemonin	(Jiao et al., 2014)

## Nephrotoxic Mechanisms

Different drugs can cause varied damage to the kidney. Hence, detection indicators are different and reflect the differentially damaged kidney parts. The damage of HM to the kidney is very complex. Thus, it is necessary to use as many indicators as possible to reflect the damage site and infer the mechanism of action. In 2008 and 2010, the U.S. Food and Drug Administration and the European Drug Administration accepted the application of PSTC and HESI and recognized eight kinds of nephrotoxic biomarkers, namely, clusterin, urinary total protein (uTP), β2-microglobulin (β2-MG), cystatin C, kidney injury molecule (Kim-1), trefoil factor-3, albumin, and renal papillaryantigen-1. These eight markers are used for drug preclinical and clinical safety evaluations. In addition, renal toxicity has many other indicators, such as NGAL, interleukin, microproteinuria, NAG, α-GST, and organic anion transporters, which can be used to detect the degree of renal injury. In this paper, according to the course of the disease and the above indicators, we divided the nephrotoxicity of HM into two categories for clarity, namely, chronic and acute toxicities. **Table 2** shows the markers that can be used to detect kidney damage.

**Table 2:** Nephrotoxic biomarkers.

Biomarkers	Injury sites	Feature	Reference(s)
clusterin	Proximal convoluted tubule; distal convoluted tubules; collecting duct	increased with high sensitivity in the preclinical AKI model, up regulated expression in AKI, renal fibrosis, renal cell carcinoma and other models	(Vinken et al., 2012)
uTP	glomerulus	predicted early glomerular injury sensitively and specifically, widely used in the prevention and monitoring of kidney diseases in clinical research	(Peterson et al., 1969)
β2-MG	Proximal convoluted tubule; glomerulus	the monomer form of it can be filtered through glomerulus and reabsorbed by proximal convoluted tubular cells; limited in clinical application due to its instability in acidic urine	(Schaub et al., 2005)
cystatin C	Proximal convoluted tubule; glomerulus; distal convoluted tubules; collecting duct	passed glomerular filtration and reabsorbed by proximal convoluted tubular cells; reflected renal tubular dysfunction due to its increase in urine	(Waring and Moonie, 2011; de Geus et al., 2012; Pianta et al., 2017)
Kim-1	Proximal convoluted tubule	used in preclinical and clinical acute renal injury studies, up regulated expression in AKI, renal fibrosis and other preclinical and clinical models	(Ichimura et al., 2008; Waring and Moonie, 2011; de Geus et al., 2012)
TFF-3	Proximal convoluted tubule	mainly expressed in intestinal and renal tissues, increased expression and activity in ulcer tissue	(Xing et al., 2013; Wang et al., 2019)
albumin	Proximal convoluted tubule; glomerulus	also increased expression in the state of inflammation, bleeding, urinary tract infections, fever or stress. not a specific index of nephrotoxicity, needs to be combined with other markers	(Schött et al., 2018; Luo et al., 2018a; Fernández et al., 2019)
RPA-1	collecting duct	predicted the early toxicity of renal papilla and has a good correlation with the degree of renal papilla injury	(Betton et al., 2012)
NGAL	renal tubule; collecting duct	considered to be a good marker of acute renal injury, and also a powerful early marker to assist the diagnosis of AKI and CKD. Increased the serum NGAL level in the state of inflammation and infection	(Waring and Moonie, 2011; de Geus et al., 2012; Avci Çiçek et al., 2016; Wang et al., 2015a)
NAG	Proximal convoluted tubule	cannot be filtered by glomeruli and increased in the urine derives from tubular damage increased expression in urine is associated with poor prognosis of AKI	(Waring and Moonie, 2011; de Geus et al., 2012)
IL-18	Proximal convoluted tubule	a cytokine with extensive immunoregulation, especially in the process of ischemic injury; considered its increased expression in urine as the early marker of AKI and the death signal of clinical critically ill patients	(de Geus et al., 2012)
L-FABP	renal tubule	considered the appearance of urinary L-FABP as a powerful sign in AKI induced by ischemic and nephrotoxin	(Yamamoto et al., 2007; de Geus et al., 2012)
IGFBP7/ TIMP2	/	related to cell cycle arrest, and proved to be useful biomarkers of AKI, but still need to be further verified	(Kashani et al., 2013; Verhagen et al., 2014; Bihorac et al., 2014; Gocze et al., 2015)

### Chronic Toxicity

#### Mechanism Related to Apoptosis

Cinnabaris contains the heavy metal mercury; the long-term use of cinnabars can cause the accumulation of mercury in the kidneys and cause kidney damage (Wang et al., 2015b). Clinically, acute renal function damage is often caused by the misuse of a large dose of cinnabaris, and chronic renal function damage is caused by taking a single or compound preparation for a long time as part of an excessive or conventional dose.

Cinnabaris can induce the apoptosis of renal tubular epithelial cells, and this pathway may be an important mechanism for subchronic nephrotoxicity. The apoptosis signaling pathway mediated by death receptor may be an important pathway for cinnabaris to induce apoptosis of renal tubular epithelial cells (Wang et al., 2015a). The application of mercury containing the drug wuwudan in large doses can cause the apoptosis of renal cells by regulating the expression levels of Bax/Bcl-2 apoptosis-related proteins (Li and Pan, 2012).

In addition to the above-mentioned mechanisms, mercury can induce apoptosis through the mitochondrial-mediated endogenous pathway. This process will increase the content of free radicals in the mitochondria, change the activity of protease on mitochondrial membrane, improve the permeability of mitochondrial membrane, affect the synthesis of ATP, and lead to the release of cytochrome C. After entering the cytoplasm, cytochrome C enhances the oxidation of cells and then induces cell apoptosis (Gao et al., 2009).

After 2 weeks, the administration of 80 μmol·L^-1^ of emodin can inhibit the proliferation of HK-2 cells, promote cell apoptosis, and decrease mitochondrial membrane potential. Its mechanism is that emodin inhibits the phosphorylation of extracellular signal regulated kinase 1/2(ERK_1/2_) and the expression of Bcl-2 in HK-2 cells. However, Bax does not undergo obvious changes, leading to the dominance of pro-apoptotic gene proteins, the development of cells towards pro-apoptosis, and the increase in the release of cytochrome c, thereby activating caspase-8 and downstream caspase-3 and causing apoptosis (Wang et al., 2010).

#### Mechanism of Oxidative Damage

The long-term administration of total alkaloids of *Leonurus ja ponicus* Houtt. leads to obvious oxidative damage and renal toxicity in rats (Sun, 2017). This condition may be caused by the large amount of reactive oxygen species (ROS) produced by the body after the drug acts on the kidney, thus directly damaging the kidney cells. The possible mechanism may be as follows: ROS reacts with the macromolecular substances of the cell membrane to form lipid peroxide, thus damaging the oxidative phosphorylation process, making the energy metabolism abnormal, and finally leading to abnormal mitochondrial function, reduced SOD and XOD secretion, cell damage, and apoptosis (Huang and Sun, 2010).

*Rheum palmatum* L. and its anthraquinone compounds can cause renal damage under certain conditions (Yan et al., 2006; Liu et al., 2017; Deng et al., 2018). The main toxic components of *R. palmatum* L. are rhein, emodin, and aloe emodin. The long-term administration of *R. palmatum* L. causes certain toxicity to the kidney of mice (Hu et al., 2019). At a dose of 0.35g · kg^-1^ · D^-1^, the toxicity is obvious, and a significant gender difference is observed. Toxicity is more obvious in males than females. The mechanisms underlying its potential toxicity may be the imbalance in the glutathione antioxidant system, the induction of excessive oxidation, the triggering of inflammatory reaction, the activation of the expression of caspase-3, and the induction of cell apoptosis.

### Cytokine Pathway

Aristolochic acid can cause renal interstitial fibrosis. The occurrence of renal interstitial fibrosis is related to the increase of extracellular matrix (ECM) synthesis and the decrease of ECM degradation. TGF-β is one of the important cytokines that promote ECM synthesis (Sun et al., 2018). The expression of TGF-β, CTGF, and ECM increase considerably in kidney tissues of patients (Zhang et al., 2005; Bai et al., 2014; Tsai et al., 2014). Exogenous aristolochic acid I could increase the mRNA expression of TGF-β and ECM in renal tubular epithelial cells and renal interstitial fibroblasts (Zhu et al., 2007; Tian et al., 2014; Lu et al., 2016; Li M. et al., 2018). The specific anti-TGF-β1 neutralizing antibody could partially block the expression of FN and HK-2 cell apoptosis induced by aristolochic acid I (Li et al., 2004). The abnormal expression of FN could promote cell proliferation, leading to renal interstitial fibrosis (Nortier et al., 2015; Chen et al., 2018; Li M. et al., 2018; Yin et al., 2018). TGF-β1 partially mediates the occurrence of FN and the apoptosis of renal tubular epithelial cells induced by aristolochic acid I.

#### Influence on Cell Transdifferentiation

Chronic AAN (CAAN) and renal interstitial fibrosis can occur in patients treated for a long time with low-dose aristolochic acid (He et al., 2008). VIM is expressed in the renal tubular epithelial cells of patients with CAAN, whereas the expression of CK decreases (Chai et al., 2009; Yi et al., 2018). This finding indicates that renal tubular epithelial cells undergo phenotypic transformation during the course of the disease and are converted into myofibroblasts.

#### Mechanism Related to Ischemic Changes

The kidney tissues of 33 patients treated with aristolochic acid showed renal ischemia, glomerular collapse and shrinkage, thickening of the capsular sac wall, and thickening of the interlobe arteries and arterioles (Depierreux et al., 1994). Many patients taking *Akebia quinata* (Thunb.) Decne. have renal ischemic changes in the pathological area (Shi et al., 2016; Wang et al., 2018). Hence, aristolochic acid may cause renal damage through vascular damage, the specific manifestation of which is the damage to the tubular wall of the kidney, thereby causing ischemia and eventually leading to renal tubular atrophy and interstitial fibrosis. After administration of 10 g/kg of *A. obliqua* S. M. Hwang to rats in the experimental group for 4 weeks, 6-keto-PGF1α and TXB2 in urine, plasma, and kidney tissue decreased remarkably (Ye et al., 2003). Both are metabolites of PGI2/TXA2. PGI2/TXA2 is among the key factors that determine renal blood flow and function. However, because of the instability of its molecular structure, the content of PGI2/TXA2 can be determined by detecting the content of 6-keto-PGF1α and TXB2. Hence, the changes in the contents of the two compounds can reflect the vascular constriction caused by the abnormal prostaglandin system, which is one of the mechanisms underlying renal injury.

### Immune-Mediated Pathway

Continuous administration of esculentoside A for 7 days causes renal injury. In this case, p-IκBα protein expression increases significantly, whereas IκBα protein expression decreases obviously. TNF-α and IL-1β expression are remarkably upregulated. Hence, the nephrotoxicity induced by esculentoside A in rats is very closely related to NF-κB. The potential nephrotoxic mechanism of esculentoside A may be the activation of the NF-κB signal, phosphorylation of the target protein of IκB α, and the overexpression of the downstream inflammatory factors TNF-α and IL-1β to induce renal damage (Yang X. et al., 2019).

#### Acute Toxicity

##### Mechanism Related to Apoptosis

Aristolochic acid induces cell apoptosis and causes direct damage to renal tubular epithelial cells, especially the proximal tubular epithelial cells (Yang H. Y. et al., 2011). Its mechanism may be related to the increase of intracellular calcium concentration (Hsin et al., 2006). By using aristolochic acid I to induce LLC-PK1 cell apoptosis model and by applying calcium antagonist (lacidepine) to reverse this effect, the calcium ion concentration caused by aristolochic acid I in the cell decreases, and the number of apoptotic cells is substantially reduced. Aristolochic acid I may cause cell apoptosis by regulating intracellular calcium concentration (Gao et al., 2000; Ma et al., 2015). In addition, the aristolochic acid metabolite, aristolochia lactam, is possibly one of the toxic components. The microscopic images of aristolochia lactam-treated HK-2 cells show typical apoptosis characteristics, in which the cell body shrinks, turns around, and even falls off (Wen et al., 2006; Jing et al., 2017).

##### Mechanism of Oxidative Damage

*T. regelii* Sprague. et Takeda. poisoning often leads to acute kidney injury and renal failure. ROS plays an important role in acute renal failure (Matsushita et al., 2011; Stratta et al., 2012; Ghosh et al., 2013; Xi et al., 2017; Hajihashemi et al., 2018). When the body’s oxidation and antioxidant functions are disordered, and a large number of oxygen free radicals cannot be promptly removed, this results in the occurrence of lipid peroxidation damage, which may be one of the nephrotoxic mechanisms of *T. regelii* Sprague. et Takeda (Huang et al., 2019). Triptolide, the main toxic components of *T. regelii* Sprague. et Takeda, can considerably inhibit the activities of SOD and GSH-Px and remarkably increase the expression of ROS and MDA in rat kidney. These changes are positively correlated with dose. Moreover, the expressions of ROS and MDA is closely related to the degree of renal injury (Yang F. et al., 2011). This finding shows that triptolide can inhibit the activity of antioxidant enzymes in the renal tissue of rats, thereby breaking the balance of oxidation and antioxidation. ROS becomes difficult to remove from renal tissues, resulting in the accumulation of ROS, which in turn leads to severe lipid peroxidation and serious kidney damage.

##### Cell Transporter Inhibition

The main nephrotoxic component of *Gardenia jasminoides* Ellis is gardenoside. The renal toxicity of gardenoside is related to the inhibition of tubule transporters (Feng et al., 2016). Geniposide can remarkably inhibit the expression of Oat1 and Oat3 in the kidney at high doses (300 mg · kg^-1^) for a short period (Feng, 2016). Oat1 and Oat3 are the main organic anion transporters that mediate the toxic substance excretion of the kidney (Nigam et al., 2015; Wen et al., 2018); their expressions are distributed in the rat kidney, especially in the renal tubules and suprarenal glands. The decrease in the expression of Oat1 and Oat3 may cause inflammation in the kidney (Masereeuw and Russel, 2010), thereby reducing the excretion of renal toxic substances and increasing the accumulation of organic anions, and leading to kidney damage.

##### DNA Damage Mechanism

**Figure 2:**
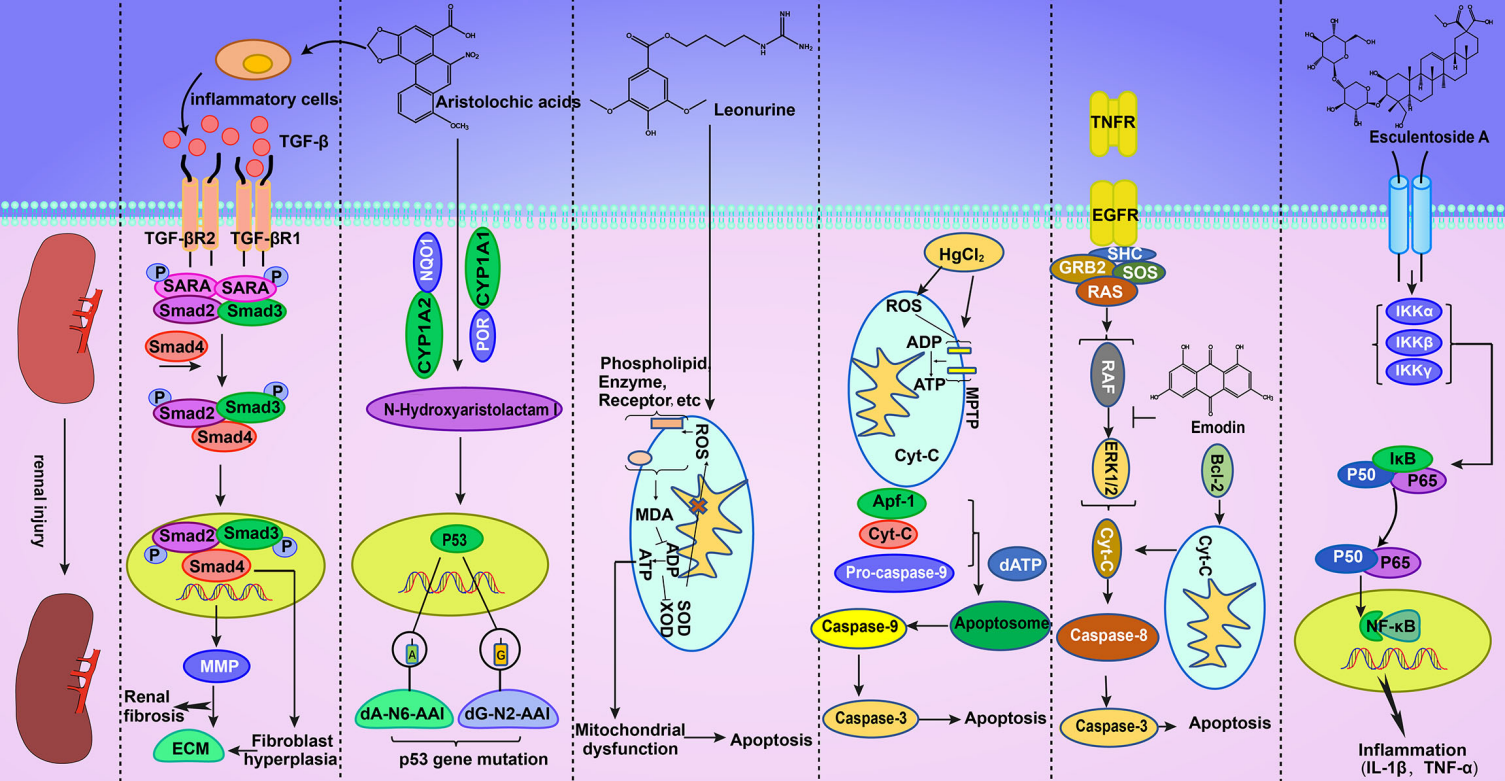
AAI can activate TGF-β/Smad-independent signaling pathways to cause abnormal cell proliferation. This effect results in the production of a large amount of ECM, which can aggravate the degree of renal fibrosis. In addition, AAI can cause *P53* gene mutations in the nucleus. Geniposide can increase ROS *in vivo*, thereby damaging oxidative phosphorylation and subsequently leading to apoptosis. Mercury can combine with biological macromolecules on the cell membrane, improve mitochondrial permeability, release Cyt-c, enhance oxidation, and cause apoptosis. Emodin can inhibit the phosphorylation of ERK1/2, thus triggering apoptosis. Esculentoside A can cause cell inflammation through the NF-κB signaling pathway.

The metabolites of aristolochic acid *in vivo*, namely, aristolochic acid I and aristolochic acid II, can covalently bind with deoxyadenine and deoxyguanine in DNA to produce DNA adducts (Jelaković et al., 2019; Yang H. Y. et al., 2019), thereby damaging the DNA structure, affecting DNA biochemical function, and causing mutations in the *P53* gene (Slade et al., 2009; Zhang et al., 2015; Sborchia et al., 2019). This adduct was first detected in the kidney of patients with CHN (Arlt et al., 2001; Stiborová et al., 2001). A certain dose of aristolochic acid I can cause DNA damage to renal tubular epithelial cells, leading to a comet tail phenomenon. Moreover, aristolochic acid I can cause cell cycle arrest in the G2/M phase (Li et al., 2006). Hence, aristolochic acid can be toxic to the kidney through the DNA damage pathway (Li et al., 2010). **Table 3** and **Table 4** summarizes some mechanisms of kidney injury. **Figure 2** gives a detailed description of some kidney injury mechanisms.

**Table 3:** Nephrotoxic Mechanism of Single Component.

Component	Experimental cell/animal	Path	References
emodin	HK-2	MAPK/ERK Signal transduction pathway	(Wang et al., 2010)
rhein	Rat	Glutathione antioxidant system	(Hu et al., 2019)
Aristolochic acid	HK-2	TGF-β/Smad independent signaling pathways (β-catenin, Ras-Raf-Erk1/2 signaling pathways)	(Li J. et al., 2018)
Aristolochic acid	HK-2	6-keto-PGF1α and TXB 2	(Ye et al., 2003)
Aristolochic acid	LLC-PK1	intracellular calcium concentration	(Gao et al., 2000)
Aristolochic acid	Rat	P38 MAKP signaling pathway	(Zhang et al., 2015)
Esculentoside A	Rat	NF-κB signaling pathway	(Yang X. et al., 2019)

**Table 4:** Nephrotoxic Mechanism of HM.

HM	Experimental animal	Path	References
Cinnabaris	Rat	Death receptor mediated apoptosis signaling pathway	(Wang et al., 2015a)
Cinnabaris	Rat	Mitochondrial mediated endogenous pathway	(Gao et al., 2009)
*Leonurus ja ponicus* Houtt.	Rat	the purine metabolism	(Huang and Sun, 2010)
*Tripterygium regelii* Sprague. et Takeda.	Rat	the purine metabolism	(Huang et al., 2019)
*Gardenia jasminoides* Ellis.	Rat	Oat1ˎ Oat3	(Masereeuw and Russel, 2010)

## Causes of Nephrotoxicity

More than 100 kinds of HM preparations that cause kidney damage are recorded in the Pharmacopoeia of the People’s Republic of China. These HM drugs are represented by *Aristolochia obliqua S. M. Hwang, Caulis Aristolochiae manshuriensis, Radix Euphorbiae pekinensis*, cinnabar, and *Fructus Meliae toosendan*. The causes of their nephrotoxicity are diverse. The use of drugs with strong renal toxicity in clinical practice should be avoided as much as possible. If the use of such drugs is unavoidable, substituting other drugs with the same efficacy or processing the drugs should be considered. Toxicity must be reduced or eliminated through reasonable processing and compatibility.

### Environmental Factors

The cultivation and growth of HM plants are inevitably affected by their surrounding environment. Large differences may exist in the compositions of the same kind of medicinal materials from different places of origin. Patients often opt to increase dosages for treatment when the content of effective ingredients is low. In this case, the toxic components ingested by patients also increase, thus resulting in kidney damage. When the soil, air, and water of the habitat of the HM plants are polluted, adverse phenomena, such as heavy metal concentrations exceeding standards, may occur (Gao and Shi, 2020).

### Misuse of HM

Homonyms and synonyms are common in HM but may represent drugs from completely different plants and with greatly different compositions. These drugs are often misused given their identical or similar names. In Belgium, cases of kidney damage caused by taking HM for weight loss (Vanherweghem et al., 1993) are due to the use of *A. obliqua S. M. Hwang* as *Stephaniae tetrandra Radix*. Although the names of the two drugs are similar, the drugs themselves are different. *A. obliqua S. M. Hwang* has a high content of aristolochic acid, which can cause a series of diseases, such as the apoptosis or necrosis of renal tubular epithelial cells and renal ischemia (Zhang et al., 2019).

### Excessive Dosage

Ancient and modern Chinese medicine practitioners have developed a series of symptomatic prescriptions during practice over the thousand years of HM use. In clinical application, prescriptions are often adjusted to increase their applicability in accordance with the needs of the disease and the differentiation and treatment of syndromes. Excessively high dosages result in the accumulation of toxic components in the body because of untimely metabolism; this effect will cause kidney damage in the long run (Wang and Liu, 2009).

### Lack of the Understanding of HM

Given the lack of the understanding of HM, users may be confused by folk traveling doctors and refer to folk prescriptions, thus resulting in the misuse or abuse of HM and ultimately in kidney damage.

### Improper Processing or Boiling

Processing has played an important role in the history of HM as a major measure for reducing the toxicity and increasing the efficiency of HM drugs. If drugs are properly processed, the toxicity of HM drugs can be greatly reduced or even eliminated, and at the same time, efficacy may be increased (Zhang et al., 2020). In addition, the selection of processing utensils, time, and temperature for the boiling process is crucial. For example, casseroles, rather than iron, aluminum, and other metal utensils, are often selected for processing and boiling HM preparations.

### Improper Compatibility

Compatibility is also a vital measure for reducing toxicity and increasing the efficiency of HM. HM drugs should not only be compatible with each other but also with Western medicine. However, inappropriate compatibility can lead to injury. For example, when Shuanghuanglian injection is used in combination with glucose, insoluble particles are produced, thus affecting metabolism and causing kidney damage (Zhang et al., 2013). *Radix Aconiti Kusnezoffii* in combination with *Radix Trichosanthis* and *Bulbus Fritillariae thunbergii* cause a series of adverse reactions (Li, 2016; Li et al., 2016).

### Special Constitution

A small number of patients with allergic constitution and special genetic diseases may not tolerate certain drugs. Intolerance results in kidney damage.

### Improper Use

Although some drugs are toxic, adverse reactions can be avoided when they are used correctly in the clinic. For example, most mineral drugs often lead to kidney damage when used internally but can exert a bacteriostatic and bactericidal effect when used externally (Bai et al., 2011; Ding, 2012; Liu, 2020).

## Clinical Application

### HM Containing Aristolochic Acid

Acute renal failure Yin Guang analyzed 31 cases of patients with AAN and found eight cases of patients with acute renal failure (ARF) caused by taking large doses of *Caulis A. manshuriensis* decoction (dose > 10 g/day). The cases exhibited renal injury accompanied by gastrointestinal or liver injury and high levels of small-molecular proteins in urine (Yin et al., 2010). In addition, 58 cases of patients with AAN were examined *via* renal puncture. Light microscopy showed that in the patients, renal tubular epithelial cells were severely denatured, necrotic, and disintegrated, and a small amount of lymphoid and mononuclear cells were sporadically infiltrated. The results of electron microscopy revealed that the surfaces of tubular epithelial cells had lost microvilli. Moreover, mitochondrial swelling was found in renal tubular epithelial cells. In addition to secondary lysosome formation, organelle disintegration and renal interstitial edema were discovered. Furthermore, the foot process of glomerular visceral epithelial cells exhibited mild segmental fusion, and the mesangial matrix increased slightly; however; electronic compacts were not observed (Chen et al., 2001).

Chronic renal failure Longdan Xiegan pill has the effects of relieving the dampness and heat of liver and gallbladder, as well as dizziness, tinnitus, and deafness. One of its prescriptions is *Caulis A. manshuriensis*, which contains a large amount of aristolochic acid that causes renal damage. A total of 21 cases of patients with chronic renal failure caused by taking Longdan Xiegan pills were analyzed. Most of the patients took the medicine for more than 1 year or even for more than 10 years (Li S. H. et al., 2018). In patients with chronic renal failure, light microscopy showed that the renal interstitium appeared multifocal or exhibited massive fibrosis, renal tubules showed multiple or large areas of atrophy, and some glomeruli presented ischemic basement membrane shrinkage and sclerosis. Electron microscopy results revealed a large number of fascicular collagen fibers in the renal interstitium, and the basement membrane of renal tubules and glomeruli had thickened and shrunk (Chen et al., 2001).

### *Tripterygium regelii* Sprague. et Takeda.

In the treatment of glomerular diseases, *T. regelii* Sprague. et Takeda. can eliminate urinary protein. Although its curative effect is exact, its toxic and side effects are severe. Six patients with nephrotic syndrome due to taking *T. regelii* Sprague. et Takeda. were observed. Among them, four developed ARF within 21–120 days after taking *T. regelii* Sprague. et Takeda. Liver and kidney functions were slightly damaged in two cases, and blood cell counts (red blood cells, white blood cells, and platelets) decreased and menstrual disorders were observed. Renal biopsy indicated severe renal interstitial and tubular lesions. Under light microscopy, obvious inflammatory cell infiltration was found in tubules and interstitium, and the obvious degeneration, necrosis, and atrophy of tubular epithelium cells were observed (Wang et al., 1996). The causes of death of 83 patients who died from taking *T. regelii* Sprague. et Takeda. were analyzed. Among them, 65 cases were due to ARF or circulatory failure (Wang et al., 1999).

### *Leonurus ja ponicus* Houtt.

*Leonurus ja ponicus* Houtt. was first published in Shennong’s Classic Materia Medica. It is considered to be a safe and nontoxic HM drug that is widely used in the clinic. It has the functions of activating blood and regulating menstruation, disinhibiting urine, dispersing swelling, clearing heat, and removing toxins. A poisoning death caused by *Leonurus ja ponicus* Houtt. was reported in the late 1980s. The patient took a 400 g decoction of *Leonurus ja ponicus* Houtt. and died of abdominal pain, headache, lower back pain, and multiple organ bleeding 1 day later. Analytical results showed that the excessive dosage and repeated use of *Leonurus ja ponicus* Houtt. resulted in renal failure and death by poisoning (Luo et al., 2018c).

### Fructus Xanthii

*Fructus Xanthii* is the fruit of *Xanthium Caulis et Folium*, which is mainly used to treat wind cold headache. A patient experienced acute liver and kidney damage and coagulation dysfunction by ingesting a large number of unprocessed *Fructus Xanthii*. Light microscopy showed that the patient’s renal tubulointerstitial lesions were severe. Moreover, in the patient, the tubular structure was clear, and diffuse renal tubular epithelial cells were swollen and degenerative. The patient exhibited occasional renal tubular atrophy and renal interstitial fibrosis with a small amount of inflammatory cell infiltration, and also presented a small number of eosinophils (Wang et al., 2013). Another patient experienced upper abdominal discomfort, nausea, vomiting, diarrhea, and reduced urine production accompanied by facial and lower limb edema; the patient was clinically diagnosed with acute renal failure and toxic hepatitis (Sun and Hei, 1997).

### Cinnabar

Cinnabar has the effect of tranquilizing the mind. It is mainly used in the treatment of palpitations, insomnia, and seizures. A man developed abdominal pain, nausea, and vomiting after taking cinnabar (4.5 g/day) for 1 month to treat palpitations and insomnia in accordance with a folk prescription. After he continued taking cinnabar for 1 month, he developed general weakness, lower-limb edema, proteinuria, and oliguria and gradually stopped producing urine. His blood urea, urea nitrogen, nonprotein nitrogen, and creatinine levels increased significantly. In addition, his liver function transaminase and blood potassium levels increased, whereas his blood chlorine and blood glucose levels decreased. He was diagnosed with mercury poisoning and acute renal failure (Guo and Li, 2001). One woman took more than 100 g of cinnabar within 30 days to treat palpitations. She developed nausea, vomiting, severe abdominal pain, and diarrhea. Her stool was brown likely because of the corrosion of gastrointestinal mucosa. On the first day after her gastrointestinal symptoms appeared, she exhibited oliguria; on the second day, she did not produce urine and her BUN was 50.5mg%. She was diagnosed with acute renal failure (Zhang and Wang, 1985).

## Prevention Strategies

Although many HMs have nephrotoxicity and their ingredients and mechanisms are quite complex, some necessary prevention measures should be employed to reduce or even avoid renal damage.

Knowledge about HM should be further disseminated, and the indications of HM should be strictly controlled. In the process of using traditional Chinese medicine, people should focus on the source of HM, processing methods, and standardized methods of use. To prevent the occurrence of drug allergy, the doctor should ask for the details on the patient’s history of drug use and allergies. The drug that once caused allergy should not be reused. Dosage and duration of medication need to be controlled within a reasonable range; this approach can prevent the accumulation of drugs in the body, which results in toxicity.

Considering that the toxic components of some HMs are also their effective ingredients, besides the abovementioned general prevention and control measures, the most effective attenuating measures for HM involve processing and compatibility.

Processing refers to the baking, cooking, frying, washing, soaking, bleaching, steaming, and boiling of Chinese HMs. Through these methods, the efficacy of HMs is enhanced, and their side effects are reduced. Moreover, the performance and efficacy of HMs are changed. Simple processing can facilitate the storage and transportation of most Chinese medicines. The processing of HMs dates back thousands of years in the past. In the “Prescriptions for Fifty-two Diseases,” several methods of processing HM are recorded for the first time. Several books on Chinese medicine, such as “Inner Canon of Huangdi,” “Sheng Nong’s herbal classic,” “Treatise on Cold Pathogenic Diseases,” and “Synopsis of Golden Chamber,” discuss the processing of HMs and have hundreds of processing methods. Processing is an effective and reliable measure for reducing toxicity and increasing efficiency, according to Chinese medicine history.

The nephrotoxicity of HMs is a serious toxic side effect of HMs. This area has been explored since its discovery. Statistics show that in the national standard, approximately 801 compound Chinese patent medicines contain rhubarb. Its application range includes multiple medical fields. It is used for detoxification, lipid lowering, and weight loss. Considering its good taste, rhubarb is used as a flavoring ingredient in Western desserts. According to the “Pharmacopoeia of the People’s Republic of China,” more than 20 kinds of processed products have been made from rhubarb. Raw, cooked, and alcohol-processed rhubarb and rhubarb charcoal are the most commonly used rhubarb artillery products in modern clinical practice. The anthraquinone component in rhubarb is its effective component, but it causes hepatotoxicity and nephrotoxicity. After processing, the effective ingredients of rhubarb change. In comparison with processed rhubarb, raw rhubarb has stronger heat clearing and detoxifying effects (Fu et al., 2014). Processing greatly reduces the anthraquinone components. After processing by wine, vinegar, and other methods, the glycosidic bond in the anthraquinone component of rhubarb is broken at high temperature, resulting in a large amount of free anthraquinone (especially physcion and chrysophanol) (Zhu et al., 2016). Subsequently, the toxicity of rhubarb, the effect of purgation, and the stimulatory effect on the gastrointestinal tract are substantially reduced.

*Aconitum* is associated with heart, nerve, and digestive system toxicity, but it has good anti-inflammatory, analgesic, immunosuppressive, anti-tumor, and cardiotonic effects. It can also lower blood pressure and dilate blood vessels and can be used to treat chronic rheumatism heart failure and chronic renal failure (Wei et al., 2019). Therefore, a high-temperature decoction is often combined with physical soaking to accelerate the decomposition of toxic alkaloids in aconitum, to maximize the drug’s clinical effect (Singhuber et al., 2009). Magnoliae Officinalis Cortex is generally clinically processed into ginger juice Magnoliae Officinalis Cortex by different methods. The contents of alkaloids and some glycosides decreased significantly after the processing of Magnoliae Officinalis Cortex; moreover, the toxicity is reduced (Yu et al., 2010). Many HMs, such as *Melia toosendan*, motherwort, and cinnamon, are used after going through various processing methods for the achievement of attenuation and synergistic effects.

Compatibility refers to the combined application of drugs to produce a certain synergistic or detoxifying effect. Chinese medicine is divided into seven kinds of laws in clinical application. In addition to the use of a single drug to treat a disease with a single condition, the six other rules refer to the description of drug compatibility. A combination of two drugs with similar efficacy can enhance the efficacy of the original drug. One medicine is the main drug, whereas the other is the auxiliary drug. When used together, the auxiliary drug can improve the efficacy of the main drug. The side effects of one drug can be inhibited by the other. One drug can eliminate the side effects or destroy the efficacy of the other drug. The use of two drugs together can also induce severe toxic side effects.

For example, *T. wilfordii* Hook F. has anti-inflammatory, analgesic, anti-tumor, and immunomodulatory effects. It is used in the treatment of immune, renal, and skin diseases; it is especially used for rheumatoid arthritis (Song et al., 2020). However, considering its severe toxic side effects, especially on the liver and kidney, the clinical application of *T. wilfordii* Hook F. has been limited (Li et al., 2014; Qu et al., 2015; Xi et al., 2017; Zhou et al., 2017). Many studies have been conducted to reduce its toxicity and expand its clinical applications. To achieve detoxification by compatibility, various detoxification measures can be employed. One method is the combination with a single medicine to reduce toxicity. After exploring the usage rules of *T. wilfordii* Hook F., various single medicines that are compatible with *T. wilfordii* Hook F. were found, including Radix Astragali, *Salvia miltiorrhiza*, Radix Paeoniae Alba, licorice, Szechuan lovage rhizome, and Radix Rehmanniae. The compatibility of *T. wilfordii* with licorice and Radix Astragali can reduce its nephrotoxicity. Second, combination with compound drugs can reduce *T. wilfordii*’s toxicity. In clinical practice, compound compatibility has been used to reduce the toxicity of *T. wilfordii* Hook F. to ensure its clinical application. Many compound medicines can effectively reduce the toxicity of *T. wilfordii* Hook F. and expand its clinical applications, such as Xinfeng Capsule, External Applied Compound *T. wilfordii* Hook F. and others. Xinfeng Capsule is compounded with Radix Astragali, Coix Seed, *T. wilfordii* Hook F., and Centipede. Xinfeng Capsule can treat arthrosis in patients with rheumatic diseases and comprehensively improve the extra-articular disease and quality of life of patients. No obvious side effects and functional damage of liver and kidney are found during the treatment. With the development of modern western medicine and technology, HMs have gradually become modernized. In terms of compatibility and toxicity reduction, new types of attenuation methods to reduce toxicity have been developed, such as combining with western medicine and studying the compatibility of ingredients. For the treatment of rheumatoid arthritis, some of the western medicines compatible with *T. wilfordii* Hook F. are methotrexate, leflunomide, and etanercept (He et al., 2014; Zhou et al., 2018). In addition, the combination of tripterysium glycoside tablets with calcium dobesilate dispersible tablets or methylprednisolone is used clinically to treat nephrotic syndrome, and adverse reactions caused by this treatment are relatively slight (Shang et al., 2018). Traditional single drug compatibility and compound drug compatibility have the disadvantages of complex ingredients, low effective content, and uncontrollable quality. Therefore, some scholars have proposed a new way to reduce toxicity, which is to study the compatibility of ingredients. The combination of total glucosides of paeony and tripterygium glycoside has increased efficiency and low toxicity in the treatment of lupus nephritis (Li et al., 2009). The combination of tripterygium glycoside and artesunate can effectively be used to treat adjuvant arthritis in rats. Diammonium glycyrrhizinate can significantly reduce the cytotoxic effect induced by *T. wilfordii* Hook F. on MDCK cells, effectively antagonize chromosomal damage, and reduce gastrointestinal reactions.

The toxicity of *M. toosendan* to the liver, kidney, and hematopoietic system of rats showed significant time–effect and dose–effect relationships (Xiang et al., 2012). Moreover, it can be used in the treatment of abdominal distention and pain. Hence, *M. toosendan* is often used in combination with Radix Paeoniae Alba to reduce liver inflammatory response. Fructus Xanthii is used in the clinical treatment of nasopharyngeal carcinoma and the pain and paralysis of joint tissue caused by rheumatism. The toxic proteins contained in Fructus Xanthii can cause damage to the parenchymal organs, especially to the liver (Wang et al., 2011). Therefore, Fructus Xanthii is often used in combination with Radix astragali to reduce its damage to the liver.

In conclusion, processing and compatibility play a large role in reducing the toxicity and increasing the efficiency of HM. Therefore, when inheriting the tradition, people should use new technology to explore new methods of reducing toxicity and increasing the efficiency of HM to improve the clinical application of HM.

## Conclusions and Future Perspectives

In this review, the nephrotoxic components in HM and their mechanism of toxicity were introduced in detail. On this basis, various causes of nephrotoxicity were proposed. The traditional measures of reducing toxicity and increasing efficiency should be used in accordance with modern research to achieve treatment.

HM was once considered as natural, nontoxic, and green. However, a large number of clinical reactions show that HM drugs contain many unknown ingredients, some of which are toxic, whereas some components may react in the human body to produce toxic substances. These situations suggest that research on the toxicity of HM should be strengthened. In addition, the metabolic process of each component in the body should be studied to prevent the accumulation of toxic drugs. In this regard, metabolomics and other technologies can be used to study and speculate the *in vivo* processes and toxicity mechanism of each component to improve studies on the prevention and control measures of HM components.

In this review, the nephrotoxicity of HM drugs was summarized in detail to ensure their safe application in clinical practice. At the same time, quality control standards for HM should be established worldwide to promote the development of the Chinese medicine industry.

## Author Contributions

XX: Conceptualization, Formal analysis, Investigation, Writing—original draft. RZ, JY, and MZ: Conceptualization, Formal analysis, Investigation. XW: Formal analysis, Investigation. KW and GC: Funding acquisition, Project administration, Validation, Writing—review. All authors contributed to the article and approved the submitted version.

## Funding

This work was financially supported by the National Natural Science Foundation of China (No.81922073), the Traditional Chinese Medicine Key Scientific Research Fund Project of Zhejiang Province (No.2018ZY004), and Natural Science Foundation Exploration project of Zhejiang Province (LQ20H280003).

## Conflict of Interest

The authors declare that the research was conducted in the absence of any commercial or financial relationships that could be construed as a potential conflict of interest.
